# The proximal experience of awe

**DOI:** 10.1371/journal.pone.0216780

**Published:** 2019-05-23

**Authors:** S. Katherine Nelson-Coffey, Peter M. Ruberton, Joseph Chancellor, Jessica E. Cornick, Jim Blascovich, Sonja Lyubomirsky

**Affiliations:** 1 Department of Psychology, The University of the South, Sewanee, Tennessee, United States of America; 2 Department of Psychology, University of California, Riverside, California, United States of America; 3 Department of Psychological and Brain Sciences, University of California, Santa Barbara, California United States of America; University of Melbourne, AUSTRALIA

## Abstract

Research on awe has grown exponentially in recent decades; however, few studies have considered whether awe-inspiring experiences also inspire other emotions. In two studies, we explored whether interventions targeting awe also evoke other discrete emotions. Additionally, we considered two constructs that may be associated with increases in each emotion—self-relevant thoughts and connectedness. In Study 1, we manipulated awe in virtual reality and examined the potential effects of a prototypical awe experience—a spacewalk accompanied by an audio clip of Carl Sagan’s *Pale Blue Dot*. In Study 2, we manipulated awe with a video depicting scenes of Earth from outer space paired with the same audio clip. Across both studies, a prototypical awe experience was associated not only with awe, but with compassion, gratitude, love, and optimism, along with connectedness and self-relevant thoughts. Furthermore, we found that increases in self-relevant thoughts and connectedness in response to the awe induction predicted increases in each emotion evoked and vice-versa. These findings suggest that experiences that are commonly considered awe-inspiring—such as viewing a picturesque landscape—may be more appropriately conceptualized more broadly as self-transcendent. More work is needed to determine whether the documented benefits of awe may be more appropriately interpreted as the benefits of self-transcendent emotions.

## Introduction

“From out there on the Moon, international politics look so petty. You want to grab a politician…drag him a quarter of a million miles out and say, ‘Look at that.’”—Edgar Mitchell.

“I felt very, very small.”—Neil Armstrong, on seeing the Earth from the Moon.

At the sight of Earth from outer space, astronauts Edgar Mitchell and Neil Armstrong likely felt intense awe—an emotion entailing the perception of vastness and the need to accommodate new information [1]. Individuals in the midst of awe describe “feeling small,” whether they are taking a spacewalk against the backdrop of Earth, feeling dwarfed by a life-size T-Rex skeleton, or simply watching the sun set over the endless ocean [2]. Research on awe has grown exponentially in recent years [3]. A PsycINFO search of the term “awe” reveals more than 500 publications since Keltner and Haidt’s [1] seminal article—more than twice the number of publications on awe prior to 2003. Such studies demonstrated that awe is associated with a host of benefits across contexts, including increased prosocial behavior [2, 4], greater exploration of the physical world [5], greater humility [6], expanded time perception [7], reduced reliance on cognitive shortcuts when processing new information [8], reduced inflammation [9], and greater overall well-being [7]. Much of this work relies on experimental inductions of awe; however, few studies have considered whether such inductions produce not just awe, but a variety of other emotions. In the current studies, we explore whether an intervention targeting awe also evokes other positive states (i.e., self-transcendent emotions, positive emotions, connectedness, humility). Furthermore, we consider two potential constructs that may be associated with increases in each emotion—self-relevant thoughts and connectedness.

### Eliciting awe

Experimental studies eliciting awe have relied on a variety of induction procedures, including writing about awe-inspiring experiences (e.g., [6, 8, 10]), viewing awe-inspiring images (e.g., [8, 11]), reading awe-inspiring stories [7], listening to awe-inspiring music [12], watching awe-inspiring videos (e.g., [2, 6, 8, 10]), and having awe-inspiring experiences (e.g., [2, 6, 13]). Following the induction procedure, these studies commonly include a measure of the emotion awe (e.g., “I felt awe, wonder, amazement”) to verify that the induction (i.e., the awe experience) elicited the emotion (i.e., awe). However, these induction procedures may elicit other emotions in addition to awe, which could contribute to effects on a variety of outcomes. In the current research, we consider whether an awe manipulation inadvertently evokes other emotions—namely, self-transcendent emotions. To distinguish the induction of awe and the emotion awe, throughout this paper, we use the term “awe experience” to refer to any induction or experience that might elicit awe, and we simply use the term “awe” to refer to awe as an emotion.

Experiments relying on writing paradigms typically provide participants with a definition of awe and ask them to describe a recent experience that evoked feelings of awe, wonder, or amazement [6, 7, 10, 14]. Others prompt participants to describe a specific prototypical awe experience, such as encountering nature [2] or a panoramic view for the very first time [8]. Experiments relying on the use of images to evoke awe typically present participants with a series of photographs of vast scenes, such as nature scenes (e.g., aurora borealis; [8]) or images of tall buildings [11]. The most common approach to eliciting awe uses short videos depicting nature scenes [2, 7, 10, 15, 16, 17] or depictions of earth from outer space [6, 7, 8]. Finally, experiential methodologies provide participants with specific experiences that would evoke awe, such as viewing an expansive T-Rex skeleton [13], viewing the surrounding area from the top of a bell tower [6], or visiting a grove of tall trees [2]. Across induction procedures, images of the universe, planet Earth, stars, or outer space are quite common: 51% of 35 experiments relied on such images to induce feelings of awe [2, 6, 7, 8, 10, 11, 13, 14, 15, 16, 17, 18, 19].

In the current studies, we elicited awe by displaying scenes from outer space via experiential (using virtual reality; Study 1) and video (Study 2) methodologies. Previous research suggested that views from outer space are particularly awe-inspiring [20], and as noted above, several studies have depicted scenes of stars or outer space to elicit awe [6, 7, 8]. In our first study, we aimed to induce a state of awe by simulating the experience of an astronaut while presenting audio clips from Carl Sagan’s *Pale Blue Dot*. Although most humans will never venture into space, advances in digital immersive environments have allowed researchers to design and immerse participants in environments that had previously been impossible. Furthermore, recent work suggests that virtual reality is a particularly effective tool for eliciting awe, given its ability to provide participants with an ecological, yet controlled environment [21]. In our second study, we elicited awe via a video depicting scenes of Earth from outer space, while also playing clips from Carl Sagan’s *Pale Blue Dot*. We selected these two approaches to elicit awe given their power to elicit emotions, their prevalence in the research literature, and the ability to present comparable experiences across two methodologies requiring very different amounts of effort to create in the laboratory.

### Emotions associated with awe experiences and their mechanisms

In addition to eliciting awe, prototypical awe experiences—such as viewing the Earth from outer space—likely elicit a range of emotions. For example, astronauts’ descriptions of their experiences in space include feeling unexpected and overwhelming emotion [20]. Awe experiences have also been characterized as self-transcendent [20], and awe is classified as a self-transcendent emotion, along with compassion, gratitude, inspiration, admiration, elevation, and love [22]. Despite plausible connections to other emotions, however, relatively few studies have considered the link between awe, and awe-eliciting experiences, and other self-transcendent emotions. In the current study, we explored potential discrete self-transcendent emotions—including gratitude love, and compassion—associated with an awe-eliciting experience. We also considered whether awe-inspiring experiences are associated with other discrete positive and negative emotions to better understand the specificity of these effects to self-transcendent emotions.

In addition, we explored two potential cognitive patterns—self-relevant thoughts and connectedness—that may be associated with each emotion evoked. Although self-transcendent emotions are hypothesized to encourage people to focus on others instead of themselves [22], and awe is associated with self-diminishment (e.g., [2]), classic appraisal theories of emotion suggest that people first appraise whether a situation is personally relevant and then experience an emotion [23, 24]. Thus, in the midst of an awe-eliciting experience, people must first think about themselves in order to adjust their own self-concepts. In the current studies, we explored the possibility that awe experiences may be perceived as self-relevant, which is in turn associated with self-transcendent emotions. For example, viewing the Earth from space is likely to be highly self-relevant (e.g., making one “feel small”), thus eliciting self-transcendent emotions, but observing the world’s largest Lego brick may have few (if any) implications for one’s self-concept. Furthermore, in our second study, we sought to distinguish self-relevance per se from the small self, given previous research linking awe to the small self (e.g., [2]).

Self-transcendent emotions are also theorized to arise in situations calling for a focus on other people, suggesting that connectedness to others may mediate the effects of self-transcendent experiences on self-transcendent emotions [22, 25]. Awe is associated with feelings of oneness with others and friends [17], as well as collective engagement [10], and astronauts’ descriptions of space travel include an increased sense of connectedness to others and to Earth overall [20]. Furthermore, previous research indicates that connectedness is associated with increases in positive emotions in general [26, 27]; however, few studies have considered connectedness as a potential antecedent of self-transcendent emotions. In the current studies, we explored whether awe-inspiring experiences elicit feelings of connectedness, which are in turn associated with each specific emotion. Notably, however, the reverse may also be true: Self-transcendent feelings elicited by awe experiences may lead to greater feelings of connectedness. Consistent with this reasoning, prior research has shown that awe experiences (e.g., seeing the earth from space) promote feelings of connectedness via self-transcendence [18, 19]; that is, awe experiences bring about feelings of awe and other self-transcendent emotions, which in turn promote greater feelings of connectedness.

### Current studies

We explored the proximal experience of awe across two studies using two methods to elicit awe. These data were collected as part of a series of studies designed to examine the link between awe and humility, which we then used to consider the hypotheses reported here. In Study 1, we manipulated awe in an immersive virtual environment (IVE; commonly called “virtual reality”) and examined the potential effects of a prototypical awe experience—a spacewalk accompanied by an audio clip of Carl Sagan’s *Pale Blue Dot*—on positive, negative, and self-transcendent emotions, along with self-relevant thoughts, connectedness, and humility. Furthermore, we examined whether increases in self-relevant thoughts and connectedness in response to the awe manipulation were associated with increases in the emotions evoked. In Study 2, we manipulated awe with a video depicting scenes of Earth from outer space paired with an audio clip of Carl Sagan’s *Pale Blue Dot*, and examined its effects on positive, negative, and self-transcendent emotions, along with self-relevant thoughts, the small self, connectedness, and humility. Additionally, we considered whether increases in self-relevant thoughts, connectedness, and the small self were associated with increases in the self-transcendent emotions evoked. Although we expected the *Pale Blue Dot* manipulations to increase self-transcendent emotions, awe, and connectedness, we approached the causal links between those variables from an agnostic, exploratory standpoint. To that end, we examined self-transcendent emotions as both outcomes (explained by connectedness and self-relevant thoughts) and mediators (leading to effects on connectedness and self-relevant thoughts), with no *a priori* expectation that either causal model will be clearly supported over the other.

## Study 1

### Method

#### Participants

Ninety-four undergraduate students (69% female, *M*_age_ = 19.97) participated in this study, advertised as “Science and VR,” in exchange for $10. The sample size was predetermined to be 100, which would provide adequate power (80%) to detect a medium effect (*r* = .40). A plurality of participants were White (48.9%), followed by Asian American (42.6%), Latino(a) (22.3%), Native American (1.1%), and Other (3.2%).

#### Procedure

Seated participants were immersed in the virtual world via an NVIS sx60 head-mounted display. While immersed, they were randomly assigned to an experimental or control condition. Experimental (“Pale Blue Dot”) participants (*n* = 47) passively moved through an open door, where they viewed the Earth from space (as if in orbit on a spaceship). As the viewer’s “spaceship” zoomed away from Earth, a narrator offered observations adapted from Carl Sagan’s *Pale Blue Dot*. Participants in the control (“Planetary Models”) condition (*n =* 47) moved to an adjacent living room environment, wherein small models of the Earth and Pluto sat on a nearby coffee table, and participants heard facts about Pluto. See S1 Supporting Information for narrator transcripts. Participants completed questionnaires at three time points: immediately before and after the experimental manipulation while still within the IVE (IVE Pre/Post-Manipulation Survey) and on a desktop computer after leaving the IVE (Non-IVE Post-Manipulation Survey). This study was approved by the Institutional Review Board at the University of California, Santa Barbara.

#### Measures

In the within-IVE survey, participants answered questions about their feelings of connectedness (i.e., “I felt closer to others and all of humanity”) and self-relevant thoughts (i.e., “I had thoughts about myself and how what was happening affected me personally”) during the simulation. Participants rated their agreement on all items by selecting a point on a line ranging from *Strongly Disagree* to *Strongly Agree*. They also completed the 6-item Brief State Humility Scale (BSHS; post-manipulation α = .78; [28]), which distinguishes humility from inflated/deflated self-regard (e.g., “I feel that, overall, I am no better or worse than the average person”). The BSHS was completed in both the pre- and post-manipulation surveys; all other measures were completed only post-manipulation.

After exiting the IVE, participants completed the Modified Differential Emotions Scale (mDES; [29]), which includes 11 positive emotions as indicated by grouped trios of related emotion descriptors (e.g., awe, wonder, amazement; glad, happy, joyful), and 9 negative emotions (e.g., angry, irritated, annoyed; ashamed, humiliated, disgraced). Each trio of descriptors formed a single item (e.g., awe was indicated by the single item “awe, wonder, amazement”). Participants rated their emotions felt during the experiment on a scale ranging from 1 (*never*) to 5 (*all the time*). In addition to considering each emotion, as well as optimism, individually, we calculated scores for overall positive emotions (α = .91) and negative emotions (α = .79), respectively. Although the self-transcendent emotions are highly inter-correlated, a confirmatory factor analysis of multi-item measures of each emotion in Study 2 revealed that they can be distinguished from one another as discrete emotions (see Results of Study 2 for details). We therefore examine each self-transcendent emotion as an independent outcome of the Pale Blue Dot manipulation. A correlation matrix of all variables, are reported in S1 Supporting Information.

### Results

#### Main effects of pale blue dot

To consider the proximal experience of awe, we began with independent samples *t*-tests comparing reports of discrete emotions, positive and negative emotions composites, as well as reported connectedness, self-relevant thoughts, and humility among participants in the Pale Blue Dot condition relative to Planetary Models (see Table 1). Participants in the Pale Blue Dot condition reported significantly greater self-transcendent emotions—that is, gratitude, compassion, optimism, awe, and love—as well as greater happiness, pride, surprise, shame, fear, and overall positive emotions. Furthermore, participants in the Pale Blue Dot condition reported greater connectedness, self-relevant thoughts, and humility. Notably, the strongest effects of the manipulation were for the self-transcendent emotions—gratitude, compassion, optimism, awe, and love. See Table 1 for means and standard deviations by condition, as well as *t*-tests and effect sizes for each comparison.

**Table 1:** Main effects of pale blue dot (via VR) on emotions, self-relevant thoughts, connectedness, and humility (Study 1).

	Pale Blue Dot (*n* = 47)	Control (*n* = 47)		
	*M* (*SD*)	*M* (*SD*)	*t*(92)	*r* [95% CI]
Gratitude	3.47 (1.23)	2.13 (1.14)	5.49***	.50 [.33, .63]
Compassion	2.85 (1.30)	1.66 (1.05)	4.89***	.45 [.28, .60]
Optimism	3.32 (1.29)	2.19 (1.04)	4.68***	.44 [.26, .59]
Awe	3.62 (1.01)	2.74 (0.97)	4.28***	.41 [.22, .56]
Love	2.38 (1.26)	1.55 (0.97)	3.57***	.35 [.16, .51]
Ashamed	1.43 (0.68)	1.09 (0.28)	3.16**	.31 [.12, .48]
Happy	3.09 (1.21)	2.34 (1.09)	3.13**	.31 [.12, .48]
Proud	2.60 (1.41)	1.87 (1.14)	2.74**	.27 [.08, .45]
Surprised	3.00 (1.25)	2.36 (1.24)	2.48*	.25 [.05, .43]
Scared	1.38 (0.85)	1.11 (0.38)	2.04*	.21 [.01, .39]
Disgust	1.30 (0.69)	1.09 (0.35)	1.89^+^	.19 [-.01, .38]
Content	3.91 (1.00)	3.57 (1.14)	1.54	.16 [.04, .35]
Interest	3.60 (1.10)	3.32 (1.11)	1.22	.13 [-.08, .32]
Sad	1.30 (0.69)	1.21 (0.41)	0.73	.08 [-.13, .27]
Contempt	1.26 (0.53)	1.19 (0.45)	0.63	.07 [-.14, .26]
Amused	2.26 (1.13)	2.13 (1.06)	0.57	.06 [-.14, .26]
Embarrassed	1.19 (0.50)	1.23 (0.48)	0.43	.04 [-.16, .24]
Angry	1.38 (0.87)	1.36 (0.64)	0.14	.01 [-.19, .21]
Guilty	1.21 (0.51)	1.21 (0.51)	0.00	.00 [-.20, .20]
Flirtatious	1.28 (0.58)	1.30 (0.75)	0.15	-.02 [-.22, .19]
Positive Emotions	3.10 (0.84)	2.35 (0.78)	4.47***	.42 [.24, .58]
Negative Emotions	1.31 (0.43)	1.19 (0.29)	1.59	.16 [-.04, .35]
Self-Relevant Thoughts	19.33 (0.21)	19.16 (0.17)	4.32***	.41 [.23, .57]
Connectedness	19.32 (0.22)	19.13 (0.15)	4.80***	.44 [.27, .60]
Humility	0.48 (0.11)	0.43 (0.11)	2.22*	.22 [.02, .40]

Note.

^*+*^*p* < .10.

*p < .05.

**p < .01.

***p < .001. Discrete emotion variables are ordered by magnitude of effect size.

#### Connectedness and self-relevant thoughts as mechanisms

**Fig 1:**
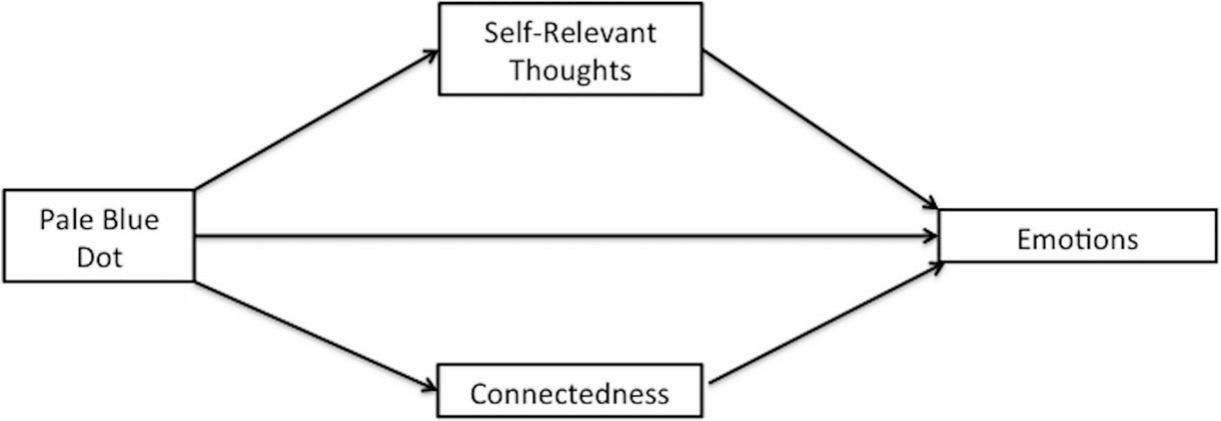
Conceptual model for indirect effects of connectedness and self-relevant thoughts on self-transcendent emotions (Study 1).

Next, we explored connectedness and self-relevant thoughts as potential mechanisms that might be related to the emotions experienced as a result of the Pale Blue Dot. Using Hayes’ [30] recommended procedures, we estimated path coefficients, as well as bootstrap bias-corrected confidence intervals (with 5,000 bootstrapped samples) for the indirect effects of Pale Blue Dot relative to Planetary Models on each emotion through connectedness and self-relevant thoughts (entered simultaneously; see Fig 1 for a conceptual model and Table 2 for a summary of results).

**Table 2:** Indirect effects of self-relevant thoughts and connectedness on emotion outcomes (Study 1).

	Unmediated Direct Effects of PBD	Mediated Direct Effects of PBD	Indirect Effects of Self-Relevant Thoughts	Indirect Effects of Connectedness
Gratitude	1.34*** [0.86, 1.83]	0.51* [0.07, 0.95]	0.27* [0.08, 0.55]	0.56* [0.29, 0.92]
Compassion	1.19*** [0.71, 1.68]	0.44 [-0.02, 0.89]	0.18 [-0.01, 0.46]	0.57* [0.28, 0.98]
Optimism	1.13*** [0.65, 1.61]	0.42 [-0.05,0.89]	0.30* [0.09, 0.64]	0.41* [0.17, 0.76]
Awe	0.87*** [0.47, 1.28]	0.46* [0.02, 0.89]	0.07 [-0.13, 0.28]	0.35* [0.12, 0.69]
Love	0.83*** [0.37, 1.29]	0.08 [-0.35, 0.50]	0.24* [0.08, .0.50]	0.52* [0.28, 0.86]
Positive Emotions	0.75*** [0.42, 1.08]	0.16 [-0.13, 0.46]	0.22* [0.08, 0.40]	0.37* [0.20, 0.62]
Negative Emotions	0.12 [-0.03, 0.27]	0.08 [-0.09, 0.25]	0.02 [-0.06, 0.09]	0.02 [-0.07, 0.12]
Humility	0.05* [0.004, 0.09]	0.04 [-0.1, 0.09]	-0.01 [-0.04, 0.01]	0.02 [-0.01, 0.06]

Note.

^*+*^*p* < .10.

*p < .05.

**p < .01.

***p < .001. This table presents a summary of analyses testing the indirect effects of self-relevant thoughts and connectedness on self-transcendent emotions, which were included as simultaneous predictors in models predicting each emotion (with emotions tested in separate models).

Analyses revealed direct effects of Pale Blue Dot on self-relevant thoughts, *b* = 0.17, *p* < .001, and connectedness to others, *b* = 0.19, *p* < .001 (*a* paths). In turn, both self-relevant thoughts and connectedness predicted greater gratitude, optimism, awe, love, and overall positive emotions, *bs >* 1.26, *p*s < .05, whereas only connectedness predicted greater compassion, *b* = 3.05, *p* < .001 (*b* path). Finally, the bias-corrected 95% confidence intervals (CIs) for the indirect effects of self-relevant thoughts on gratitude, optimism, love, and overall positive emotions did not contain zero. Similarly, the bias-corrected 95% CIs for the indirect effects of connectedness on gratitude, compassion, optimism, awe, love, and positive emotions also did not contain zero. Pairwise contrasts indicated that the indirect effects of self-relevant thoughts and connectedness did not significantly differ for any emotion outcome (*b*s < 0.39, *p*s > .05). Table 2 presents a summary of all indirect effects and 95% CIs.

#### Connectedness and self-relevant thoughts as outcomes

**Fig 2:**
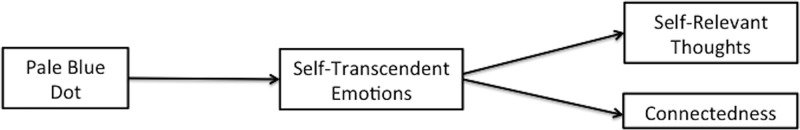
Conceptual model of indirect effects of self-transcendent emotions on self-relevant thoughts and connectedness (Study 1).

To test the reverse-causal hypothesis, we examined additional path models with self-relevant thoughts and connectedness as outcomes separately, and self-transcendent emotions as mediators (entered separately; see Fig 2 for a conceptual model and Table 3 for a summary of results.)

**Table 3:** Indirect effects of self-transcendent emotions on self-relevant thoughts and connectedness (Study 1).

	Gratitude	Compassion	Optimism	Awe	Love	Positive Emotions	Negative Emotions	Humility
Self-Relevant Thoughts	0.10* [0.05, 0.15]	0.07* [0.03,0.12]	0.08* [0.04, 0.13]	0.04* [0.001, 0.08]	0.06* [0.02, 0.11]	0.09* [0.04, 0.14]	0.01 [-0.01, 0.02]	-0.004 [-0.02,0.02]
Connectedness	0.12* [0.07, 0.19]	0.11* [0.06, 0.16]	0.09* [0.04, 0.14]	0.06* [0.03, 0.11	0.08* [0.03, 0.13]	0.10* [0.05, 0.16]	0.01 [-0.01, 0.02]	0.01 [-0.01, 0.04]

Note.

*p < .05. This table presents a summary of analyses testing the indirect effects of self-transcendent, positive, and negative emotions on self-relevant thoughts and connectedness. All self-transcendent emotions were tested in separate models.

### Discussion

In sum, we found that watching the Earth zoom away as if on a spaceship, while hearing a narrator read Carl Sagan’s *Pale Blue Dot* in an immersive environment, elicited self-transcendent emotions (awe, compassion, gratitude, love), and optimism. Our manipulation also elicited greater happiness, pride, surprise, shame, fear, and overall positive emotions. Notably, however, a pattern emerged such that the manipulation elicited self-transcendent emotions most strongly. Furthermore, this experience led people to feel more connected to others and increased self-relevant thoughts, which partially explained, and were explained by, relative increases in self-transcendent and positive emotions, but not negative emotions. The strengths of this study include the use of a novel virtual environment to induce a variety of emotions and states, and the consistency of the indirect effects of self-relevant thoughts and connectedness to others on self-transcendent emotions and vice-versa. However, these findings need replication given the small sample, limited measures, and exploratory nature of the findings. Our second study replicated these findings in a larger sample with extended measures. In addition, given previous research linking awe to a small sense of self [2, 13, 31], we sought to distinguish self-relevant thoughts from the small self. Finally, instead of experiencing the Pale Blue Dot in an immersive environment, participants in Study 2 watched an analogous video on a personal screen.

## Study 2

### Method

#### Participants

Participants (*N* = 172; 39% female) were recruited from Prolific Academic to participate in a study about “Science and Emotions” in exchange for $2.50. The majority were White (75%), followed by Asian American (10.5%), Latino(a) (5.8%), African American (3.5%), More than One (2.9%), and no response (2.3%). Participants’ ages ranged from 18 to 72, with an average age of 34.55 (*SD* = 11.32). Prior to data collection, we decided to recruit approximately 200 participants to maximize power. Data collection continued until 201 participants responded to our study; however, 29 failed an attention check (“Please select the option ‘Somewhat disagree’ below”) and were thus excluded from analyses (including the demographic numbers reported above).

#### Procedure

Participants were randomly assigned to watch one of two short videos. Similar to Study 1, the first video depicted Earth zooming away (as if aboard a spaceship) while a narrator read selections from Carl Sagan’s *Pale Blue Dot* (Pale Blue Dot condition *n* = 83). The second video depicted images of Earth from space and provided information about the Earth’s rotation and orbit around the sun (Earth Facts condition *n* = 89). After watching their assigned video, participants completed measures of self-transcendent emotions, other-connectedness, self-relevant thoughts, small self, humility, and other positive and negative emotions. Prior to data collection, we pre-registered this study on the Open Science Framework (osf.io/svy3c) to test a set of hypotheses generated by an earlier set of findings regarding the associations between awe, humility, connectedness, and self-relevant thoughts; however, because our specific hypotheses were not supported, we do not present the pre-registered analyses here. This study was approved by the Institutional Review Board at the University of California, Riverside.

#### Measures

Participants responded to 12 items measuring 4 *self-transcendent emotions* (adapted as single items from the mDES; [29]): awe (i.e., awe, wonder, amazement; α = .84), gratitude (i.e., grateful, appreciative, thankful; α = .91), love (i.e., love, closeness, trust; α = .88), and compassion (i.e., compassion, sympathy, concern; α = .83). Because the *Pale Blue Dot* manipulation strongly increased optimism in Study 1, we also assessed optimism (i.e., optimistic, hopeful, encouraged; α = .93) alongside the self-transcendent emotions, giving a total of 15 items in the self-transcendent emotion measure. Participants reported the extent to which they experienced each emotion while watching the video on a scale ranging from 1 (*not at all*) to 5 (*the entire time*). To verify that the self-transcendent emotions (including optimism) could be differentiated from one another, we conducted a confirmatory factor analysis (CFA) with the three items from each self-transcendent emotion loaded onto their respective factors. Fit of the 5-factor model was acceptable, TLI = .94, RMSEA = .086 (95% CI = [.069, .102]), SRMR = .042. Additionally, each of the 15 items had loadings of at least .61 on their respective factors, and only two items (“Awe” and “Concern”) had loadings below .75. The CFA therefore provided sufficient evidence to analyze the self-transcendent emotions as discrete variables rather than as a single composite.

Participants completed 6 items measuring *connectedness* to others and humanity adapted from the connectedness subscale of the Balanced Measure of Psychological Needs [32]. Participants responded to each item (e.g., “I felt close and connected with all of humanity”) regarding their feelings while they were watching the video on a scale ranging from 1 (*no agreement*) to 5 (*much agreement*). Cronbach’s α was .96.

Participants responded to 5 items reflecting private self-consciousness (e.g., “I am thinking about myself a lot”; adapted from [33]) on a scale ranging from 1 (*strongly disagree*) to 7 (*strongly agree*). These 5 items were combined to reflect *self-relevant thoughts*, Cronbach’s α = .92.

In addition, participants responded to 5 items reflecting the *small self* (or self-diminishment; e.g., “I feel small or insignificant”; [2]) on a scale ranging from 1 (*strongly disagree*) to 7 (*strongly agree*). These 5 items were combined to create a composite of the small self, Cronbach’s α = .92.

As in Study 1, participants completed the BSHS (α = .82) regarding their current feelings of *humility*.

Finally, participants completed 18 additional items measuring *positive emotions* (i.e., happy, pleased, joyful, enjoyment/fun, content, serene, peaceful, amused, silly, glad, interested, alert, curious, proud, confident, self-assured, surprised, astonished) and 19 items measuring *negative emotions* (i.e., worried/anxious, angry/hostile, frustrated, depressed/blue, unhappy, irritated, annoyed, ashamed, humiliated, disgraced, disgust, embarrassed, self-conscious, blushing, sad, downhearted, scared, fearful, afraid; adapted from the mDES [29], and from the Affect Adjective Scale [34]). Participants rated their current feelings of each emotion on a scale ranging from 1 (*not at all*) to 7 (*extremely*). Our results focus on the effects of our manipulation on discrete positive and negative emotions in addition to composite positive (α = .96) and negative (α = .92) emotions. Correlations among all variables are reported in S1 Supporting Information.

### Results

#### Main effects of pale blue dot

As in Study 1, we conducted *t-*tests comparing the experiences of those who watched the Pale Blue Dot video relative to the Earth Facts video. Watching a short video depicting images of Earth as if from a spaceship led to increases in all five self-transcendent emotions, as well as connectedness, self-relevant thoughts, and small self, relative to control (see Table 4 for means, standard deviations, *t*-tests, and effect sizes). Examination of the confidence intervals surrounding the effect sizes reveals that the Pale Blue Dot video elicited greater compassion than gratitude, awe, and love, but not optimism, self-relevant thoughts, connectedness, or the small self.

**Table 4:** Main effects of pale blue dot (via video) on emotions, self-relevant thoughts, connectedness, and humility (Study 2).

	Pale Blue Dot (*n* = 83)	Control (*n* = 89)		
	*M (SD)*	*M (SD)*	*t*(170)	*r* [95% CI]
Compassion	3.21 (0.98)	2.10 (1.07)	7.14***	.48 [.36, .59]
Optimism	3.13 (1.07)	2.52 (1.27)	3.38**	.25 [.11, .39]
Gratitude	3.39 (1.02)	2.92 (1.28)	2.68**	.20 [.05, .34]
Awe	3.71 (0.96)	3.33 (1.00)	2.60*	.20 [.05, .34]
Love	2.85 (0.99)	2.39 (1.28)	2.58*	.19 [.05, .33]
Disgust	1.69 (1.19)	1.30 (0.80)	2.49*	.18 [.04, .33]
Fear	1.79 (1.16)	1.55 (1.10)	1.40	.11 [-.04, .25]
Worried	2.78 (1.53)	2.52 (1.46)	1.17	.09 [-.06, .24]
Sad	2.14 (1.16)	1.94 (1.14)	1.11	.08 [-.07, .23]
Shame	1.53 (0.82)	1.40 (0.77)	1.02	.08 [-.07, .23]
Happy	3.37 (1.10)	3.33 (1.86)	0.88	.07 [-.08, .21]
Depressed	2.30 (1.49)	2.13 (1.52)	0.73	.06 [-.09, .20]
Content	3.96 (1.33)	3.79 (1.80)	0.72	.06 [-.10, .20]
Pleased	3.70 (1.56)	3.53 (1.90)	0.64	.05 [-.10, .20]
Pride	3.44 (1.55)	3.27 (2.00)	0.60	.05 [-.10, .19]
Interested	4.44 (1.10)	4.27 (1.73)	0.43	.03 [-.12, .18]
Amused	2.25 (1.28)	2.16 (1.49)	0.42	.03 [-.12, .18]
Anger	1.82 (1.19)	1.67 (1.04)	0.41	.03 [-.12, .18]
Embarrassed	1.88 (1.09)	1.72 (1.06)	0.33	.03 [-.13, .17]
Surprised	2.33 (1.61)	2.40 (1.82)	0.26	-.02 [-.17, .13]
Frustrated	1.93 (1.36)	2.01 (1.36)	0.40	-.03 [-.18, .12]
Enjoyment	3.14 (1.65)	3.26 (1.91)	0.42	-.03 [-.18, .12]
Positive Emotions^a^	3.56 (1.09)	3.44 (1.62)	0.56	.04 [-.11, .19]
Negative Emotions^b^	1.90 (0.82)	1.73 (0.78)	1.43	.11 [-.04, .25]
Self-Relevant Thoughts	4.06 (1.31)	3.18 (1.69)	3.79***	.28 [.14, .41]
Connectedness	3.16 (0.91)	2.45 (1.12)	4.56***	.33 [.19, .46]
Small Self	5.29 (1.44)	4.28 (1.61)	4.34***	.32 [.17, .44]
Humility	5.04 (1.17)	5.10 (1.17)	0.35	.03 [-.12, .18]

Note.

*p < .05.

**p < .01.

***p < .001. Discrete emotion variables are ordered by magnitude of effect size.

^a^The positive emotions composite includes the following discrete emotions: happy, content, pleased, pride, interested, amused, surprised, and enjoyment.

^b^The negative emotions composite includes the following emotions: disgust, fear, worried, sad, shame, depressed, anger, embarrassed, and frustrated.

#### Self-relevant thoughts and connectedness as mechanisms

Next, as in Study 1, we explored connectedness and self-relevant thoughts as indirect effects of the Pale Blue Dot on Self-Transcendent Emotions. Furthermore, to disentangle the role of self-relevance per se from thoughts about the small self specifically, we included reports of small self as an indirect effect. Again, using Hayes’ [30] recommended procedures, we estimated path coefficients, as well as bootstrap bias-corrected confidence intervals (with 5,000 bootstrapped samples) for the indirect effects of Pale Blue Dot relative to Earth Facts (Control) on each self-transcendent emotion through connectedness, self-relevant thoughts, and the small self (see Table 5 for a summary of all indirect effects).

**Table 5:** Indirect effects of self-relevant thoughts, connectedness, and small self on emotion outcomes (Study 2).

	Unmediated Direct Effects of PBD	Mediated Direct Effects of PBD	Indirect Effects of Self-Relevant Thoughts	Indirect Effects of Connectedness	Indirect Effects of Small Self
Compassion	1.12* [0.81, 1.42]	0.62* [0.38,0.86]	0.10* [0.02, 0.25]	0.43* [0.24, 0.66]	-0.04 [-0.15, 0.05]
Optimism	0.61* [0.25, 0.96]	0.07 [-0.20,0.35]	0.15* [0.04, 0.33]	0.48* [0.27, 0.76]	-0.10 [-0.24, 0.02]
Gratitude	0.48* [0.13, 0.83]	-0.11 [-0.38, 0.16]	0.15* [0.04, 0.33]	0.43* [0.24, 0.72]	0.01 [-0.11, 0.14]
Awe	0.39* [0.09, 0.68]	-0.06 [-0.32, 0.20]	0.08 [-0.02, 0.24]	0.15* [0.03, 0.34]	0.22* [0.10, 0.41]
Love	0.45* [0.11, 0.80]	-0.12 [-0.37, 0.13]	0.17* [0.06, 0.35]	0.51* [0.28, 0.78]	-0.10 [-0.24, 0.001]
Positive Emotions	0.12 [-0.30, 0.54]	-0.51* [-0.83, -0.18]	0.19* [0.05, 0.37]	0.57* [0.31, 0.86]	-0.13* [-0.29, -0.01]
Negative Emotions	0.17 [-0.07, 0.42]	0.01 [-0.24, -0.26]	0.12* [0.02, 0.24]	0.05 [-0.05, 0.15]	0.004 [-0.09, 0.08]
Humility	-0.06 [-0.42, 0.29]	0.03 [-0.33, 0.39]	-0.14 [-0.31, 0.01]	-0.17 [-0.36, 0.005]	0.22* [0.07, 0.40]

Note.

*p < .05. This table presents a summary of analyses testing the indirect effects of self-relevant thoughts, connectedness, and small self on self-transcendent emotions, which were included as simultaneous predictors in models predicting each emotion (with emotions tested in separate models).

Consistent with our findings from Study 1, we found a direct effect of Pale Blue Dot on connectedness, *b* = 0.71, *p* < .001, self-relevant thoughts, *b* = 0.80, *p* = .0002, and the small self, *b* = 1.01, *p* < .001 (*a* paths). Reflecting the paths from mechanisms to outcomes (*b* paths), connectedness predicted greater compassion, optimism, gratitude, awe, and love, *b*s > 0.21, *ps <* .01; self-relevant thoughts predicted greater compassion, optimism, gratitude, and love, *b*s > 0.12, *p*s < .05, and marginally greater awe, *b* = 0.09, *p* = .08; and the small self predicted greater feelings of awe, *b* = 0.22, *p* < .001, lower levels of love, *b* = -0.10, *p* = .03, and marginally lower levels of optimism, *b* = -0.10, *p* = .055.

Finally, the bias-corrected 95% CIs for the indirect effects of connectedness on compassion, optimism, gratitude, awe, and love did not include zero; the bias-corrected 95% CIs for the indirect effects of self-relevant thoughts on compassion, optimism, gratitude, love, and disgust did not include zero; and the bias-corrected 95% CIs for the indirect effects of the small self on awe did not include zero (see Table 5). Pairwise contrasts of the indirect effects revealed that connectedness more strongly predicted gratitude, compassion, optimism, and love than self-relevant thoughts and small self (*bs* > 0.29, *p*s < .05); self-relevant thoughts more strongly predicted compassion, optimism, and love (but not gratitude, *b* = 0.14, *p* > .05) than did small self (*b*s > 0.14, *p*s < .05); and the indirect effects of connectedness, small self, and self-relevant thoughts on awe did not significantly differ *b*s < 0.14, *p* > .05. In sum, connectedness partially explained increases in all self-transcendent emotions in response to Pale Blue Dot; self-relevant thoughts partially explained increases in all self-transcendent emotions except awe; and the small self partially explained increases in awe only.

#### Self-relevant thoughts and connectedness as outcomes

To test the reverse-causal hypothesis, we examined additional path models with self-relevant thoughts, connectedness, and the small self as outcomes separately, and self-transcendent emotions as mediators (entered separately; see Table 6 for a summary of results.)

**Table 6:** Indirect effects of self-transcendent emotions on self-relevant thoughts, connectedness, and small self (Study 2).

	Compassion	Optimism	Gratitude	Awe	Love	Positive Emotions	Negative Emotions	Humility
Self-Relevant Thoughts	0.89* [0.61, 1.23]	0.43* [0.18, 0.72]	0.35* [0.10, 0.61]	0.26* [0.06, 0.48]	0.36* [0.09, 0.62]	0.07 [-0.19, 0.31]	0.10 [-0.03, 0.28]	0.02 [-0.10, 0.13]
Connectedness	0.77* [0.54, 1.01]	0.35* [0.15, 0.55]	0.29* [0.08, 0.49]	0.19* [0.05, 0.35]	0.29* [0.07, 0.50]	0.06 [-0.15, 0.26]	0.06 [-0.02, 0.15]	0.01 [-0.06, 0.09]
Small Self	0.51* [0.24,0.83]	0.19* [0.05,0.38]	0.22* [0.05, 0.43]	0.31* [0.08, 0.58]	0.16* [0.03, 0.34]	0.03 [-0.08, 0.15]	0.05 [-0.02, 0.16]	-0.01 [-0.08, 0.05]

Note.

*p < .05. This table presents a summary of analyses testing the indirect effects of self-transcendent, positive, and negative emotions, as well as humility, on self-relevant thoughts, connectedness, and small self. All indirect effects were tested in separate models.

### Discussion

Mirroring the results of Study 1, we found in Study 2 that the Pale Blue Dot—experienced via video instead of virtual reality—predicted increases in self-transcendent emotions (compassion, gratitude, awe, optimism, and love), as well as disgust. Furthermore, we found that similar psychological mechanisms (self-relevant thoughts and connectedness to others) were associated with relative increases in self-transcendent emotions among participants who experienced the Pale Blue Dot. However, as in Study 1, our data were also consistent with the reverse causal path: Self-transcendent emotions predicted self-relevant thoughts and connectedness as outcomes of the Pale Blue Dot experience. Furthermore, we found that the small self was uniquely associated with increases in awe, distinguishing a specific type of self-relevance linked with awe relative to other self-transcendent emotions. The effects of the Pale Blue Dot on humility, overall positive emotions, and shame were smaller in Study 2 than in Study 1. To gain a more comprehensive understanding of the effects of Pale Blue Dot on all emotion outcomes, we meta-analytically combined the effect sizes across the two studies.

## Meta-analysis of Studies 1 and 2

In two studies using two different methods to elicit awe, we found that awe experiences were associated not only with awe, but with a range of discrete self-transcendent, positive, and negative emotions, along with connectedness and self-relevant thoughts. To gain a more accurate estimate of the effect size for the influence of the Pale Blue Dot on our outcomes, we meta-analytically combined the effect sizes from the two studies and conducted analyses using both fixed effects and random effects models [35]. The fixed effects model is statistically powerful and appropriate for small-sample meta-analyses [35, 36]. For these analyses, one-tailed *p-*values from each study were converted to *Z* scores and then combined using the Stouffer method [37]. The random effects model is notably less powerful, but allows for generalization to studies beyond this sample. For these analyses, one-sample *t-*tests were conducted on the average Fisher *Z*_*r*_ effect sizes [35, 36].

Weighted *r* effect sizes ranged from .10 (surprised) to .47 (compassion), and the effects for all five self-transcendent emotions, as well as connectedness and self-relevant thoughts, were significant using the fixed effects approach. Using the random effects approach, only the effects for compassion and disgust were statistically significant, and the effects for optimism, connectedness, and self-relevant thoughts were marginally significant (*p*s < .10; see Table 7 for a summary of our meta-analytic findings). Our analyses using the random effects model only had 1 degree of freedom, making significant meta-analytic effects difficult to detect unless the effect sizes from individual studies are nearly equal. Thus, across these two studies using similar but distinct methodologies, we found extremely consistent effects of viewing the Pale Blue Dot on self-transcendent emotions, connectedness, and self-relevant thoughts.

**Table 7:** Meta-analysis of Studies 1 and 2 (*k* = 2, total *N* = 267).

	Weighted *r* Effect Size	Unweighted *r* Effect Size	Significance Tests			
Variable	Mean [95% CI]	Mean [95% CI]	Fixed Effects Model		Random Effects Model	
			*Z*	*p*	*t*(1)	*p*
Gratitude	.31 [.20, .42]	.36 [-.61, .90]	5.47	< .001	2.17	.14
Compassion	.47 [.37, .56]	.47 [.37, .55]	7.50	< .001	26.53	.01
Optimism	.32 [.21, .42]	.35 [-.31, .78]	5.47	< .001	3.35	.09
Awe	.28 [.16, .39]	.31 [-.39, .78]	4.70	< .001	2.74	.11
Love	.25 [.13, .36]	.27 [-.26, .68]	4.13	< .001	3.22	.10
Disgust	.18 [.06, .30]	.19 [.15, .21]	3.06	.001	37.40	.01
Ashamed	.16 [.04, .28]	.20 [-.51, .75]	2.91	.002	1.66	.17
Happy	.16 [.04, .27]	.19 [-.53, .76]	2.29	.01	1.56	.18
Proud	.13 [.01, .25]	.16 [-.50, .71]	2.33	.01	1.44	.19
Surprised	.10 [-.02, .22]	.14 [-.54, .71]	1.54	.06	1.17	.23
Scared	.15 [.03, .26]	.16 [-.16, .45]	2.41	.008	3.14	.10
Connectedness	.38 [.27, .48]	.40 [-.07, .72]	6.43	< .001	5.46	.06
Self-Relevant Thoughts	.33 [.22, .43]	.35 [-.11, .68]	5.57	< .001	4.89	.06
Humility	.10 [-.02, .22]	.13 [-.45, .63]	1.26	.10	1.31	.21

## General discussion

In two studies, we explored the proximal experience of awe, finding that a prototypical awe experience—viewing Earth from outer space—was associated not only with awe, but with compassion, gratitude, love, and optimism, as well as with shame, fear, and disgust. Our awe experience also led to greater connectedness and self-relevant thoughts, which predicted increases in each self-transcendent emotion evoked and vice-versa; however, the small self uniquely predicted increases in awe in Study 2. The effects of awe experiences on self-transcendent emotions from our two studies—using immersive and video methodologies, respectively—were remarkably consistent; indeed, the top five emotions evoked (compassion, optimism, gratitude, awe, love) across the two studies were identical. Results of our internal meta-analysis confirmed this consistency, with moderate effect sizes of the awe experience on self-transcendent emotions, connectedness, and self-relevant thoughts.

### The nature of awe experiences

People experience awe in the midst of anything perceived as larger than the self (i.e., vast) that challenges current mental structures (i.e., requiring accommodation; [1]). The results of the studies presented here add to a growing body of research investigating the outcomes of awe experiences [16, 20, 38]. Our findings suggest that awe experiences (including experimental inductions of awe) are not only awe-inspiring, but that they may also give rise to a range of positive states, including several discrete self-transcendent emotions and positive emotions in general, as well as self-relevant thoughts and connectedness to others. Much like Layous and colleagues [39] found that gratitude is not the only or strongest outcome of a gratitude induction, we found that awe is not the only or strongest outcome of an awe induction. Indeed, in both of our studies, we found that compassion was the strongest emotion elicited in response to our manipulation, and compassion was elicited more strongly than awe in our second study. Notably, however, both of our studies relied on text from the *Pale Blue Dot* to elicit awe. Thus, more research is needed to consider whether the effects of this manipulation would generalize to other awe manipulations (e.g., nature videos). If additional studies replicate our findings, then experiences that are commonly considered awe-inspiring—such as viewing a picturesque landscape—may be more appropriately conceptualized more broadly as self-transcendent (see [25], for a review of self-transcendent experiences) and generically positive. More research is needed to determine whether the documented benefits of awe may be more appropriately interpreted as the benefits of self-transcendent or positive emotions.

Interestingly, our awe manipulation also elicited the negative states of shame and fear (Study 1), as well as disgust (Study 2), in addition to the positive states described above. These negative emotions may have resulted from our specific manipulations of awe in the two studies. The text of the *Pale Blue Dot* used in our studies refers to both natural disasters and abuses of power among world leaders (e.g., Adolf Hitler)—subjects that are likely to evoke these negative emotions. In addition, in our first study, participants viewed Earth from outer space in virtual reality, as if they were an astronaut traveling the solar system—an experience that may have been fear-inducing. Notably, however, the effects of these negative emotions were small (*r*s < .17) and inconsistent across the two studies (although their meta-analytic combination was significant). Future research considering potential negative emotions resulting from self-transcendent experiences would be illuminating. For example, prior work suggests that people may feel awe in response to negative events, such as powerful natural disasters or terrorist attacks [2, 38]; however, little research has considered the broader self-transcendent nature of these negative events. Such events may similarly give rise to feelings of compassion or gratitude, as people consider opportunities to help others in response to these events. For example, gratitude and love were two of the most frequently reported emotions following the September 11^th^ attacks [40].

These results have important implications for investigations of awe. For example, prior research has revealed awe to be associated with increased generosity, humility, and overall well-being [2, 4, 6, 7]. An important step in this line of research would be to consider whether our findings generalize to alternative manipulations of awe. If all awe manipulations also elicit other emotions, the patterns found in prior investigations could be due to feelings of awe or due to other emotions elicited. Notably, experiments on the effects of awe commonly contrast awe manipulations with inductions of other positive emotions, such as pride [2], amusement [2, 6, 10], or happiness [13]; however, the findings presented here suggest that, to fully understand its unique effects, researchers may wish to contrast awe with other self-transcendent emotions, such as compassion or gratitude, which also have been linked with generosity, humility, and well-being (e.g., [41, 42, 43, 44]).

### The roles of connectedness and self-relevant thoughts

We also found that awe experiences elicited self-relevant thoughts and connectedness, which were in turn associated with increases in most self-transcendent emotions. These findings offer an important contribution to research investigating the mechanisms that may give rise to the experience of discrete emotions. Together, these findings suggest that focusing on oneself and focusing on others are not mutually exclusive and that people may simultaneously contemplate the personal relevance of an event while considering their connections with others.

Although our findings regarding self-relevant thoughts may seem inconsistent with prior findings that self-transcendent emotions encourage other-focus [22], they indicate that considerations of the self may be a first step in adjusting one’s self-concept and directing one’s attention outward. Consistent with prior research, in our second study, we measured the small self as well as self-relevant thoughts and found that the *Pale Blue Dot* video led to increased awe, which in turn predicted increases in the sense of a small self. The inverse was also true: The awe manipulation predicted increases in the small self, which in turn uniquely predicted increases in awe, suggesting that awe and feelings of small self may mutually influence one another. Notably, although all self-transcendent emotions elicited by the awe manipulation were linked with self-relevant thoughts in general, only awe was related to small self. This finding offers one way to distinguish the awe-inspiring components of awe experiences (i.e., as involving self-diminishment), relative to other self-transcendent emotions. Furthermore, our findings regarding self-relevant thoughts are consistent with appraisal theories of emotion [23, 24], which suggest that in the midst of potentially emotion-inducing experiences, people must first consider the personal relevance of the situation prior to the experience of an emotion. If a situation is not deemed to be personally relevant, then it would not give rise to an emotion. Thus, self-relevance is a necessary condition for the experience of emotions.

In addition, we found that increased feelings of connectedness in response to the awe manipulation were associated with improvements in all self-transcendent emotions. These findings highlight the social nature of self-transcendent emotions. Self-transcendent emotions are often described as “other-praising” emotions [45], and distinct from other positive emotions, they commonly arise in situations calling for a focus on other people [22]. Furthermore, our findings are consistent with the robust literature linking social connectedness to improved positive emotions and overall well-being [26, 27, 46]. As with self-relevance, our studies suggest a reciprocal causal relationship between self-transcendent emotions and connectedness: People who feel connected with others may feel self-transcendent, but self-transcendent feelings may also promote subsequent feelings of connectedness. Finally, the indirect effects of positive emotions on self-relevant thoughts, connectedness, and the small self were relatively stronger in our second study. These larger effects may be a result of the use of improved measures of all outcomes in Study 2; however, more research is needed to verify these effects and their sizes.

### Strengths, limitations, and future directions

The strengths of these studies involve the use of an experimental approach to understand whether a prototypical awe experience elicits a range of discrete emotions. Whereas most prior awe experiments focused exclusively on manipulating awe alongside manipulations of a few comparison emotions [2, 6, 10], the current studies measured multiple emotions as potential outcomes of awe experiences, thus contributing understanding of the many emotions that might be produced by awe experiences. In addition, our findings were remarkably consistent across the two studies, lending confidence to the conclusions drawn here. Finally, we employed two methods for emotion induction—virtual reality (an intensive and high-cost approach) and video (a commonly used, accessible, and scalable approach). The consistency of our findings across the two methods offers validation of using videos to induce emotions.

Despite these strengths, our findings should be considered in light of a few limitations, which suggest directions for future research. First, both of our studies were conducted at a single time-point and did not consider how the effects of awe experiences unfold over time. Future research considering the long-term effects of awe experiences would be beneficial. For example, perhaps awe experiences have longer-lasting effects on awe than on other self-transcendent emotions. Additionally, longitudinal studies could demonstrate the temporal order of the mechanisms (i.e., self-relevant thoughts and connectedness) and outcomes (i.e., emotions) studied here. In light of previous research indicating that positive emotions promote social bonds [22, 47], longitudinal studies could establish, for example, whether connectedness precedes or follows self-transcendent emotions. In addition, longitudinal studies could also illuminate the temporal order of self-transcendent emotions. For example, perhaps these manipulations elicit awe, which then triggers other self-transcendent emotions. Although self-transcendent emotions can be distinguished from one another, they are nevertheless highly correlated, and testing the directionality of their associations presents a compelling direction for future research.

Second, both of our studies elicited awe by depicting scenes of Earth from outer space. The similarity of our methods across the two studies allowed us to demonstrate the direct replicability of our findings; however, future research using different awe manipulations (e.g., videos of natural beauty) to corroborate the effects reported here would be informative. For example, eliciting awe via a nature video may not promote comparable levels of connectedness as in the current studies, given the social references present in the *Pale Blue Dot*. Also, Carl Sagan’s *Pale Blue Dot* audio clip arguably contains text that directs listeners how to feel (e.g., “Our planet is a lonely speck in the great enveloping cosmic dark”), and future investigators might consider removing the audio narration. Moreover, future research contrasting awe inductions with inductions of other self-transcendent emotions would illuminate the unique effects of awe relative to these other comparable states. Considerations of the downstream consequences of awe experiences via awe and other self-transcendent emotions would be informative. For example, such studies could isolate whether increases in generosity following an awe experience (e.g., [2]) are due to increases in feelings of awe or due to increases in other self-transcendent emotions (e.g., gratitude).

Finally, the analyses reported here involved a secondary analysis of studies designed to test a separate hypothesis. Although we developed the theoretical framework presented here prior to re-analyzing the data, and we believe that that framework provides a valid basis for the hypotheses tested in this paper, further work replicating and extending these findings is needed to provide unequivocally a priori support for our hypotheses. In particular, future research should directly parse the causal interplay among self-transcendent emotions, self-relevance, and connectedness. Prior research has established that self-transcendent feelings such as awe mediate experimental effects on connectedness and self-relevance [15, 20]. Our data are consistent with these findings, but they also show that the inverse causal direction—that self-relevance and connectedness promote self-transcendence—may be correct as well. This finding suggests a possible reciprocal causal relationship between self-transcendent emotions and feelings of connectedness or self-relevance, but because our causal models were exploratory, our data cannot provide clear support for either causal path. Hence, future studies could thus test these competing hypotheses using an a priori approach.

### Concluding thoughts

People feel awe in response to a variety of experiences—viewing a beautiful sunset across a panoramic landscape, witnessing the birth of a child, or standing in the presence of a powerful, charismatic leader. Our work suggests that not only might people feel awe, wonder, and amazement in response to such events, but also gratitude, love, optimism, and compassion—feelings that may elicit, and be strengthened by, a renewed perspective on oneself and a stronger feeling of connection with others.

## Supporting information

S1 Supporting Information (DOCX)
