# Supplementary material for: The proximal experience of awe
Source: PLoS One. 2019 May 23;14(5):e0216780. doi: 10.1371/journal.pone.0216780 (PMC6532958; doi:10.1371/journal.pone.0216780)
Supplement: S1 Supporting Information — (DOCX) [file pone.0216780.s001.docx]

**Supporting Information**

**S1 Appendix. Virtual Reality Simulation Script**

***USER INSTRUCTIONS***

Please walk over to the computer terminal to complete a few survey questions.

Click on the lower screen to indicate your response to each question.

I just opened the doors for you. Please walk outside for a moment.

I’ve added some objects to the room. If you turn around you will be able to see them.

The doors are open again. Please go back inside.

Please return to the computer to complete a few more survey questions.

That concludes the simulation. Thanks for your participation. Please follow the instructions of your research assistant.

***SEQUENCE #1 – Designed to elicit a sense of awe***

*[Standing on a platform of a space ship viewing the Earth from orbit]*

As you can see, we’re a little far from home. Astronauts call it The Blue Marble. That is our home.

We’re going to take a little trip. But don’t worry, we’ll come back shortly.

*[After zooming far away from Earth]*

From this distant vantage point, the Earth might not seem of any particular interest.

But for us, it's different.

Consider again that dot. That's here. That's home. That's us.

On it everyone you love,

Everyone you know,

Everyone you ever heard of,

Every human being who ever was,

Lived out their lives.

The aggregate of our joy and suffering,

Thousands of economic doctrines, ideologies, and religions

Every hunter and forager,

Every hero and coward,

Every creator and destroyer of civilization,

Every king and peasant,

Every young couple in love,

Every mother and father, hopeful child,

Inventor and explorer,

Every teacher of morals,

Every corrupt politician,

Every "superstar,"

Every "supreme leader,"

Every saint and sinner in the history of our species lived there –

On a mote of dust suspended in a sunbeam.

The Earth is a very small stage in a vast cosmic arena.

Think of the rivers of blood spilled by all those generals and emperors so that in glory and triumph they could become the momentary masters of a fraction of a dot.

Think of the endless cruelties visited by the inhabitants of one corner of this pixel on the scarcely distinguishable inhabitants of some other corner.

How frequent their misunderstandings,

How eager they are to kill one another,

How fervent their hatreds.

Our posturings, our imagined self-importance, are all challenged by this point of pale light.

Our planet is a lonely speck in the great enveloping cosmic dark.

Ok, let’s go back home.

*[The ship returns to Earth—it zooms closer until we’re in close orbit again]*

***SEQUENCE #2 – Designed only as a comparison experience – Informational feel***

I want to give you some information about our solar system.

Pluto is the second-largest dwarf planet in the Solar System. It is smaller than the largest known dwarf planet, Eris. Its formal name is 134340 Pluto. The dwarf planet is the tenth-largest body that moves around the Sun. At first, Pluto was called a planet. Now, it is considered the second largest of the bodies in the Kuiper belt *[Rhymes with "viper"].*

Like other members of the Kuiper belt, Pluto is mainly made of rock and ice. It is quite small. It is about a fifth of the weight of the Earth's Moon and only a third of its volume. It has an odd orbit that is very sloped. The orbit of Pluto takes it from 4.4 and 7.4 billion kilometers from the Sun. This causes Pluto to sometimes go closer to the Sun than Neptune.

Since it was discovered in 1930, Pluto was thought to be the Solar System's ninth planet. In the late 1970s, the minor planet 2060 Chiron *[pronounced KAI-RUN]* was found and people learned that Pluto had a small mass. They asked why it was a major planet from then on because it was really small. Later, in the early 21st century, the scattered disc object Eris and other objects like Pluto were discovered. Eris is 27% more massive than Pluto. On August 24, 2006, the International Astronomical Union gave a definition to the word "planet" for the first time. By this definition, Pluto was not a planet anymore. It became a "dwarf planet" along with Eris and Ceres. After this, Pluto was put on the list of minor planets. A number of scientists continue to hold that Pluto should be classified as a planet.

**S1 Table 1. Correlation Matrix of All Dependent Variables in Study 1.**

|  |  | 1 | 2 | 3 | 4 | 5 | 6 | 7 | 8 | 9 | 10 | 11 |
| --- | --- | --- | --- | --- | --- | --- | --- | --- | --- | --- | --- | --- |
| 1 | Connectedness |  |  |  |  |  |  |  |  |  |  |  |
| 2 | Self-relevant thoughts | .52*** |  |  |  |  |  |  |  |  |  |  |
| 3 | Awe | .50*** | .36*** |  |  |  |  |  |  |  |  |  |
| 4 | Gratitude | .68*** | .56*** | .70*** |  |  |  |  |  |  |  |  |
| 5 | Love | .63*** | .51*** | .53*** | .73*** |  |  |  |  |  |  |  |
| 6 | Compassion | .65*** | .49*** | .55*** | .68*** | .72*** |  |  |  |  |  |  |
| 7 | Optimism | .58*** | .54*** | .56*** | .77*** | .66*** | .60*** |  |  |  |  |  |
| 8 | Amused | .26* | .35*** | .19 | .23* | .33*** | .25* | .34*** |  |  |  |  |
| 9 | Content | .26* | .21* | .56*** | .55*** | .36*** | .33** | .48*** | .18 |  |  |  |
| 10 | Joyful | .44*** | .50*** | .55*** | .63*** | .61*** | .58*** | .63*** | .45*** | .55*** |  |  |
| 11 | Interest | .35*** | .25* | .49*** | .34*** | .36*** | .33** | .36*** | .30** | .41*** | .56*** |  |
| 12 | Proud | .49*** | .53*** | .39*** | .62*** | .63*** | .53*** | .64*** | .30** | .37*** | .66*** | .43*** |
| 13 | Flirtatious | .13 | .22* | .15 | .22* | .36*** | .31** | .19 | .23* | .00 | .24* | .10 |
| 14 | Surprise | .41*** | .29** | .64*** | .56*** | .44*** | .45*** | .47*** | .21* | .37*** | .54*** | .42*** |
| 15 | Angry | -.04 | -.03 | -.21* | -.18 | -.10 | -.06 | -.26* | -.15 | -.46*** | -.25* | -.31** |
| 16 | Ashamed | .21* | .18 | .12 | .27** | .21* | .34*** | .13 | .12 | -.03 | .13 | .05 |
| 17 | Contempt | .25* | .19 | .07 | .17 | .09 | .28** | .02 | .12 | -.09 | .02 | -.01 |
| 18 | Disgust | .24* | .24* | .01 | .15 | .12 | .24* | .02 | .01 | -.08 | .02 | -.02 |
| 19 | Embarrassed | -.07 | .08 | -.01 | -.02 | -.03 | .07 | -.07 | .09 | -.08 | .11 | .12 |
| 20 | Guilty | .09 | .02 | .05 | .17 | .14 | .18 | .00 | .16 | .04 | .10 | .04 |
| 21 | Sad | -.04 | -.05 | -.13 | -.07 | -.08 | .01 | -.18 | -.05 | -.28** | -.17 | -.17 |
| 22 | Fear | .14 | .15 | .18 | .20 | .27** | .31** | .07 | .13 | .07 | .20 | .07 |
| 23 | Positive Emotions | .66*** | .58*** | .76*** | .86*** | .80*** | .76*** | .82*** | .46*** | .63*** | .84*** | .61*** |
| 24 | Negative Emotions | .15 | .14 | .00 | .12 | .11 | .25* | -.06 | .07 | -.20 | .01 | -.07 |

**S1 Table 1 (continued)**

|  |  | 12 | 13 | 14 | 15 | 16 | 17 | 18 | 19 | 20 | 21 | 22 | 23 |
| --- | --- | --- | --- | --- | --- | --- | --- | --- | --- | --- | --- | --- | --- |
| 12 | Proud |  |  |  |  |  |  |  |  |  |  |  |  |
| 13 | Flirtatious | .26** |  |  |  |  |  |  |  |  |  |  |  |
| 14 | Surprise | .37*** | .11 |  |  |  |  |  |  |  |  |  |  |
| 15 | Angry | -.08 | .02 | -.14 |  |  |  |  |  |  |  |  |  |
| 16 | Ashamed | .14 | .21* | .19 | .34*** |  |  |  |  |  |  |  |  |
| 17 | Contempt | .12 | .20 | .11 | .35*** | .55*** |  |  |  |  |  |  |  |
| 18 | Disgust | .14 | .26* | .07 | .36*** | .69*** | .47*** |  |  |  |  |  |  |
| 19 | Embarrassed | .11 | .11 | .13 | .16 | .24* | .21* | .25* |  |  |  |  |  |
| 20 | Guilty | .18 | .14 | .16 | .18 | .46*** | .41*** | .39*** | .25* |  |  |  |  |
| 21 | Sad | -.09 | .03 | -.14 | .55*** | .34*** | .49*** | .25* | .11 | .37*** |  |  |  |
| 22 | Fear | .12 | .08 | .27** | .22* | .24* | .26* | .16 | .34*** | .23* | .34*** |  |  |
| 23 | Positive Emotions | .75*** | .28** | .69*** | -.26** | .22* | .12 | .09 | .05 | .15 | -.16 | .24* |  |
| 24 | Negative Emotions | .11 | .19 | .11 | .66*** | .73*** | .71*** | .68*** | .47*** | .61*** | .69*** | .56*** | .07 |
| *Note. *p* < .05. ***p* < .01. ****p* < .001. | | | | | | | | | | | | | |

**S1 Table 2. Correlation Matrix of All Dependent Variables in Study 2.**

|  |  | 1 | 2 | 3 | 4 | 5 | 6 | 7 | 8 | 9 | 10 | 11 |
| --- | --- | --- | --- | --- | --- | --- | --- | --- | --- | --- | --- | --- |
| 1 | Connectedness |  |  |  |  |  |  |  |  |  |  |  |
| 2 | Self-relevant thoughts | .67*** |  |  |  |  |  |  |  |  |  |  |
| 3 | Small self | .53*** | .47*** |  |  |  |  |  |  |  |  |  |
| 4 | Awe | .50*** | .46*** | .53*** |  |  |  |  |  |  |  |  |
| 5 | Gratitude | .70*** | .59*** | .39*** | .66*** |  |  |  |  |  |  |  |
| 6 | Love | .74*** | .62*** | .32*** | .56*** | .83*** |  |  |  |  |  |  |
| 7 | Compassion | .73*** | .59*** | .40*** | .52*** | .70*** | .77*** |  |  |  |  |  |
| 8 | Optimism | .69*** | .58*** | .30*** | .59*** | .80*** | .85*** | .69*** |  |  |  |  |
| 9 | Happy | .59*** | .48*** | .22** | .43*** | .56*** | .63*** | .49*** | .56*** |  |  |  |
| 10 | Pleased | .60*** | .49*** | .20** | .35*** | .58*** | .65*** | .46*** | .57*** | .83*** |  |  |
| 11 | Pride | .54*** | .46*** | .13 | .27*** | .50*** | .61*** | .44*** | .57*** | .72*** | .73*** |  |
| 12 | Content | .52*** | .43*** | .26*** | .38*** | .53*** | .60*** | .42*** | .56*** | .77*** | .75*** | .67*** |
| 13 | Interest | .53*** | .41*** | .31*** | .52*** | .53*** | .55*** | .50*** | .46*** | .62*** | .56*** | .64*** |
| 14 | Amuse | .53*** | .49*** | .12 | .28*** | .47*** | .59*** | .42*** | .54*** | .66*** | .64*** | .64*** |
| 15 | Surprise | .48*** | .44*** | .23** | .43*** | .45*** | .56*** | .39*** | .45*** | .56*** | .59*** | .55*** |
| 16 | Enjoyment | .56*** | .51*** | .15* | .31*** | .53*** | .67*** | .45*** | .58*** | .80*** | .79*** | .76*** |
| 17 | Disgust | .11 | .17* | .06 | -.06 | .05 | .08 | .11 | .04 | -.06 | -.02 | .04 |
| 18 | Fear | .24** | .22** | .20* | .21** | .25*** | .25*** | .31*** | .14 | .10 | .08 | .05 |
| 19 | Worry | .28*** | .27*** | .25** | .20** | .25*** | .19* | .32*** | .19* | .03 | .05 | -.08 |
| 20 | Sad | .13 | .21** | .13 | .05 | .05 | .09 | .16* | .01 | -.18* | -.10 | -.05 |
| 21 | Shame | .34*** | .36*** | .22** | .17* | .31*** | .33*** | .34*** | .29*** | .20** | .21** | .22** |
| 22 | Depressed | -.02 | .05 | .10 | -.02 | -.01 | -.09 | -.01 | -.14 | -.28*** | -.19* | -.26*** |
| 23 | Angry | .08 | .13 | -.03 | -.16* | .01 | .01 | .04 | -.01 | -.18* | -.11 | .04 |
| 24 | Embarrassed | .38*** | .42*** | .20** | .19* | .38*** | .41*** | .37*** | .39*** | .31*** | .30*** | .34*** |
| 25 | Frustrated | -.02 | .02 | -.03 | -.12 | -.06 | -.10 | -.06 | -.11 | -.29*** | -.20** | -.12 |
| 26 | Positive Emotions | .65*** | .55*** | .24** | .45*** | .62*** | .73*** | .54*** | .65*** | .88*** | .86*** | .88*** |
| 27 | Negative Emotions | .27*** | .32*** | .18* | .08 | .22** | .22** | .27*** | .15* | -.02 | .03 | .07 |

**S1 Table 2 (continued)**

|  |  | 12 | 13 | 14 | 15 | 16 | 17 | 18 | 19 | 20 | 21 | 22 |
| --- | --- | --- | --- | --- | --- | --- | --- | --- | --- | --- | --- | --- |
| 12 | Content |  |  |  |  |  |  |  |  |  |  |  |
| 13 | Interest | .61*** |  |  |  |  |  |  |  |  |  |  |
| 14 | Amuse | .58*** | .46*** |  |  |  |  |  |  |  |  |  |
| 15 | Surprise | .49*** | .47*** | .56*** |  |  |  |  |  |  |  |  |
| 16 | Enjoyment | .76*** | .60*** | .73*** | .60*** |  |  |  |  |  |  |  |
| 17 | Disgust | -.10 | -.16* | .14 | .12 | .01 |  |  |  |  |  |  |
| 18 | Fear | .05 | .07 | .20** | .36*** | .10 | .36*** |  |  |  |  |  |
| 19 | Worry | -.11 | -.02 | .04 | .12 | -.02 | .21** | .54*** |  |  |  |  |
| 20 | Sad | -.20** | .02 | .08 | .22** | -.06 | .45*** | .54*** | .46*** |  |  |  |
| 21 | Shame | .17* | .09 | .40*** | .36*** | .25** | .61*** | .57*** | .35*** | .40*** |  |  |
| 22 | Depressed | -.33*** | -.14 | -.10 | .00 | -.21** | .30*** | .50*** | .50*** | .66*** | .35*** |  |
| 23 | Angry | -.24** | -.15* | .13 | .01 | -.08 | .56*** | .32*** | .29*** | .43*** | .43*** | .43*** |
| 24 | Embarrassed | .23** | .20** | .47*** | .40*** | .35*** | .42*** | .45*** | .33*** | .43*** | .68*** | .27*** |
| 25 | Frustrated | -.39*** | -.23** | -.07 | -.11 | -.19* | .41*** | .34*** | .42*** | .44*** | .30*** | .60*** |
| 26 | Positive Emotions | .85*** | .77*** | .77*** | .72*** | .89*** | -.01 | .15 | -.02 | -.04 | .29*** | -.23** |
| 27 | Negative Emotions | -.11 | -.02 | .25*** | .28*** | .06 | .64*** | .76*** | .61*** | .76*** | .75*** | .70*** |

**S1 Table 2 (continued)**

|  |  | 23 | 24 | 25 | 26 |  |
| --- | --- | --- | --- | --- | --- | --- |
| 23 | Angry |  |  |  |  |  |
| 24 | Embarrassed | .26*** |  |  |  |  |
| 25 | Frustrated | .72*** | .20* |  |  |  |
| 26 | Positive Emotions | -.09 | .39*** | -.25** |  |  |
| 27 | Negative Emotions | .71*** | .66*** | .67*** | .08 |  |
| *Note. *p* < .05. ***p* < .01. ****p* < .001. | | | | | | |
